# Chronicity and physical activity levels independently associate with spatial patterns of multifidus fat infiltration in chronic low back pain patients

**DOI:** 10.1007/s00586-025-09660-9

**Published:** 2025-12-26

**Authors:** Karim Khattab, Lucas K. Dziesinski, Jiamin Zhou, Aaron Scheffler, Aaron J. Fields, Conor W. O’Neill, Jeffrey C. Lotz, Jeannie F. Bailey

**Affiliations:** 1 Department of Orthopaedic Surgery, University of California, San Francisco, USA; 2 Department of Epidemiology and Biostatistics, University of California, San Francisco, USA

**Keywords:** Chronic low back pain, Multifidus, Fat infiltration, Chronicity, Physical activity

## Abstract

**Purpose:**

This study sought to identify spatial patterns of elevated fat infiltration (FI) in the multifidus associated with chronic low back pain (cLBP) chronicity (duration) and physical activity levels.

**Methods:**

On 223 cLBP patients, we mapped the FI distribution in the multifidus at L4L5 and L5S1 using 3T MRI and advanced sequences (IDEAL). Using statistical parametric mapping, we identified FI patterns independently associated with increased cLBP chronicity and decreased physical activity levels. Then, we used linear mixed-effects modeling to identify associations between cLBP chronicity, physical activity level and two FI measures, the FI% of the entire muscle cross-sectional area (overall FI%) and the FI% in the deepest 15% of the MF (deep15 FI%).

**Results:**

Increased cLBP chronicity associated with elevated FI in the deepest 24% of the MF at L4L5 (*p* < .01) and in the superficial region of the MF at L4L5 (*p* < .05) and L5S1 (*p* < .05). Lower levels of physical activity associated with higher MF FI in the deepest 10% of the MF at L4L5 (*p* < .05). We also find that increased cLBP chronicity (*p* < .01) and lower physical activity levels (*p* < .05) associated with elevated deep15 FI%, independent of age, disc degeneration, and pain severity.

**Conclusion:**

Increased cLBP chronicity and lower physical activity levels independently associate with elevated MF FI. Further, these associations are independent of age, disc degeneration, and pain severity. Lastly, physical activity may have an independent association with MF FI specific to the deep MF, where fat infiltration has been shown to associate with cLBP symptoms and adjacent disc degeneration.

## Introduction

Standard clinical imaging of chronic low back pain (cLBP) patients often identifies advanced degeneration of the intervertebral disc [1], but the relationship between disc degeneration and cLBP symptoms is often unclear. This may not be surprising since the disc is just one passive element of a complex spine stabilization system disrupted in cLBP patients [2]. The paraspinal muscles work synergistically with the discs to actively stabilize the spine, and can also be degenerated in cLBP patients [3]. The combined degeneration of active and passive spine stabilizers diminishes postural stability and is thought to increase injury risk and pain severity [4]. However, the relationship between the degeneration of the spine stabilizers and the duration (chronicity) and frequency of cLBP symptoms is yet to be established.

The multifidus (MF) is a deep paraspinal muscle (PSM) uniquely positioned to segmentally stabilize the spine and particularly degenerated in cLBP patients [5]. Fatty infiltration (FI) is associated with loss of skeletal muscle strength and is best measured from advanced MRI sequences [6]. Different structural and functional changes to the multifidus are observed in acute, recurring, and chronic LBP [5]. In recurrent LBP patients, higher PSM FI associates with a higher frequency of LBP episodes and, even in remission, these patients have higher PSM FI at lower lumbar levels than healthy controls [7]. The causal direction of these findings is unclear but suggests that either poor muscle quality is a risk factor for cLBP chronicity (duration) or, conversely, a result of cLBP.

Multifidus FI is positively associated with the presence of cLBP symptoms and adjacent disc degeneration [3, 8, 9]. Recent spatial analysis of MF FI shows that, adjusting for age, sex, and BMI, this association is specific to the deep MF at L4L5 and L5S1 [10]. However, skeletal muscle FI (myosteatosis) progresses with age and is not unique to cLBP patients or the PSMs [11, 12]. Age-related muscle degeneration and sex-specific FI differences have been observed in skeletal muscle [6] and specifically in the MF in healthy adults [13]. Lifestyle factors like sedentarism and inactivity are also implicated in the progression of myosteatosis [12, 14] and have been identified as cLBP risk factors [15, 16]. While a recent study suggests physical activity may slow age-related skeletal muscle FI [17], another study found that differences in physical activity did not explain elevated PSM FI in recurrent LBP patients [7], questioning the relevance of activity-based interventions targeting muscle quality in cLBP patients. Although inactivity is a risk factor for both cLBP and myosteatosis, it is unclear how activity levels and MF FI relate to cLBP chronicity. The interplay of these factors in cLBP is poorly understood and it is unclear if chronicity and physical activity levels play separate or interconnected roles in the relationship between muscle FI and cLBP.

We hypothesize that cLBP chronicity and lower physical activity levels individually associate with higher MF FI at the lower lumbar levels, independent of disc degeneration and pain severity. To investigate this, we mapped the spatial distribution of MF FI and identified FI patterns associated with chronicity and physical activity levels. Then, we used linear mixed-effects modeling to test for relationships between multi-level (L4L5, L5S1) MF FI and chronicity and physical activity levels. We limit our analysis to the lower lumbar levels since prior work has shown that FI measures at the upper lumbar levels are strongly associated with age, sex, and BMI, whereas FI measures at the lower lumbar levels are strongly associated with cLBP symptoms and adjacent spine features [10]. Improving our understanding of the relationship between cLBP chronicity and spatial patterns of MF FI may provide important context for exploring causal mechanisms of MF FI and its role in the presence and duration of cLBP symptoms. Further, clarifying the relationship between physical activity and spatial patterns of MF FI, independent of symptom severity and chronicity is an important step towards determining the relevance of physical activity as a targeted intervention for patients with poor muscle quality.

## Methods

### Data collection and recruitment

With IRB approval and informed consent, we recruited 223 cLBP patients from the Back Pain Consortium (BACPAC) Research Program comeBACK cohort [18], which required subjects to be above 18 years old and report low back pain for at least three of the past six months (See [18] for additional exclusion criteria). Of 230 patients with advanced MRI sequences, seven lacked physical activity and chronicity survey data, resulting in the final 223 patient cohort. Patients underwent a 3 T multi-echo MRI and completed demographic and pain surveys immediately before the scan. Pain intensity was measured on the NRS scale. Chronicity was patient-reported and measured as months since cLBP onset. Physical activity was measured as weekly MET minutes, calculated from the International Physical Activity Questionnaire (IPAQ) [19].

### MRI scans, pathology scoring, and muscle segmentation

Lumbar spine scans were performed on a 3 T scanner with standard clinical T1- and T2- weighted sequences and advanced sequences to assess disc and muscle quality. Iterative decomposition of water and fat with echo asymmetry and least-squares estimation (IDEAL) [4]) was used for fat infiltration quantification. Sequence specifications are presented in prior publications [4, 9, 20, 21]. Disc degeneration was graded at L4L5 and at L5S1 by experienced clinicians from T1 and T2 standard clinical sequences using Pfirrmann Grade [21]. We also calculated the mean disc degeneration as the average Pfirrmann Grade at L4L5 and at L5S1. In prior studies, using the same scanner, protocol, and sequence, the inter-rater reliability of Pfirrmann grading was k = 0.74. At L4L5 and L5S1, the right and left multifidus were manually segmented from T1- and T2-axial images across two slices taken from the sagittal midpoint of the disc. Segmentations were then performed on IDEAL images using co-registered T1- and T2-weighted images as a guide to help ensure accuracy at the tissue interfaces (see [4] for segmentation details). MF fat maps were then created from the segmented IDEAL images. Segmentations did not include the epimuscular fat region. During disc grading and muscle segmentation, raters were blinded to all patient demographic and survey data.

### Fat-mapping the multifidus

The full fat-mapping workflow is presented in a prior publication [9]. For each subject, we approximated the center of rotation (CoR) of each motion segment [22] then defined non-overlapping regions of interest (ROI) radiating outward at two pixel increments from the CoR. Within each ROI, we calculated the mean FI percent, creating a fat distribution curve depicting MF FI% moving radially through the muscle, deep to superficial, away from the CoR. The x-axis was distance normalized to 0–100% of the radial muscle width, with 0% and 100% representing the deepest and most superficial points of the muscle, respectively. For repeatability, fat-maps were created from the average fat-map at two axial slices at each disc level, averaging the right and left multifidus, resulting in one fat-map at each lumbar level. Fat-maps were created using Python (Python Software Foundation, www.python.org).

In addition, we measured the fat percent of the entire muscle cross-sectional area (overall FI%) and the average fat percent in the deepest 15% (from the vertebral CoR) of the MF (deep15 FI%). The inter-rater reliability of the overall FI% was k = 0.92.

### Statistical data analysis

We used statistical parametric mapping (SPM) to identify spatial patterns in fat distribution associated with physical activity levels and chronicity. We conducted multivariable regression tests at L4L5 and L5S1 using SPM general linear models accounting for age, sex, and BMI. For each SPM analysis, we calculated the SPM t-statistic (SPM{t}) at every point from 0 to 100% of the fat-maps. Then, we calculated the critical threshold test statistic (α = 0.05) to identify regions of significance. The size of these regions (clusters) correlates with the strength of significance. All SPM analysis was performed using the SPM1D package in Python, which uses random field theory to control for multiple comparisons in SPM.

Next, we created two multivariable linear regression models with cLBP chronicity as the outcome. First, to test the relationship between overall MF quality, disc degeneration, and chronicity, we used the mean overall MF FI% and the mean disc degeneration at L4L5 and L5S1 as predictors, in addition to age, sex, and BMI. Second, we repeated the analysis but replaced mean overall MF FI% with the mean deep15 MF FI% at L4L5 and L5S1 to compare associations between metrics.

Then, to test the relationship between pain severity, chronicity and physical activity level we created a multivariable linear regression model with physical activity level as the outcome. We used chronicity, pain severity, age, sex, and BMI as predictors.

Lastly, we used linear mixed-effects models to investigate multilevel (L4L5,L5S1) MF FI and its relationship to chronicity, physical activity level, pain severity, and adjacent disc degeneration. To adjust for repeated measures, subject ID was included as a random effect. In the first model, we used the deep15 MF FI% as the outcome with chronicity, adjacent disc degeneration, pain severity, and physical activity level as fixed effects, in addition to age, sex, BMI, and lumbar level. We then repeated this analysis but with overall MF FI% as the outcome, to compare the association of fixed effects with overall and deep15 MF FI%.

## Results

### Population data

Across the 223 patients (103 M, 120 F), the average chronicity was 11.9 years (±12.7) and the average MET minutes per week was 3164.8 (±2920.4) with no differences in age, BMI, chronicity, or physical activity levels between males and females (Table 1).

**Table 1:** Average age, BMI, pain intensity, cLBP chronicity, and physical activity levels in males and females

	Males, *n* = 103		Females, *n* = 120	
	Mean	SD	Mean	SD
Age	51.4	14.7	54.4	15.9
BMI	26.2	3.8	26.0	4.8
Pain Intensity	4.4	1.8	4.5	2.0
cLBP chronicity (yrs)	11.2	12.4	12.4	12.9
Physical activity (MET min/wk)	3519.6	3161.7	2862.8	2674.4

### Modeling cLBP chronicity and its relationship to multifidus fat infiltration

**Fig. 1:**
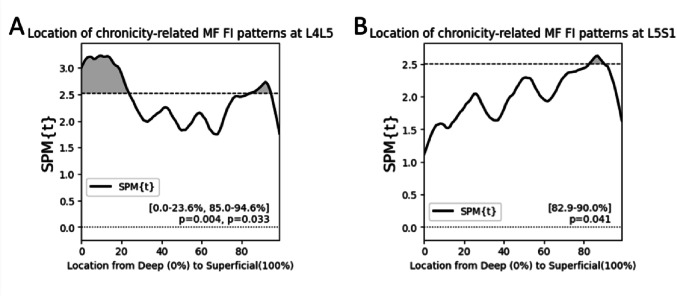
SPM general linear modeling shows patterns of FI in the deep and superficial MF associated with increased cLBP chronicity. SPM general linear models adjusting for age, sex, and BMI. Regions of significance (clusters) are shaded and represent regions in the MF where increased chronicity is associated with elevated MF FI. A cluster is specified as a region in the MF where the SPM{t} statistic (black line) crosses the critical threshold (dotted line). (**A**) At L4L5, longer cLBP duration is associated with elevated MF FI specifically in the deepest 25% of the MF and in the superficial MF. (**B**) At L5S1 longer cLBP duration is associated with elevated MF FI only in the superficial MF

Using an SPM general linear model, adjusting for age, sex, and BMI, we found that chronicity (longer duration of cLBP) associated with elevated FI in two regions of the MF at L4L5: in the deepest 24% of the muscle (*p* <.01) and in the superficial region of the MF from 85% to 94.6% of the muscle (*p* <.05) (Fig. 1A). At L5S1, this relationship was only significant in one region of the superficial MF from 82.9% to 90% of the muscle (*p* <.05) (Fig. 1B).

Using multivariable linear regression modeling of chronicity we observed that, in the overall FI% model, longer duration of cLBP associated with higher mean disc degeneration (*p* <.01) and higher mean overall MF FI% (*p* <.001) but not with age, sex, or BMI (Table 2). In the deep15 FI% model of chronicity, longer cLBP duration associated with higher mean disc degeneration (*p* <.01), higher mean deep15 MF FI% (*p* <.01), and older age (*p* <.01), but not with sex, or BMI (Table 2).

**Table 2:** Multivariable linear regression of cLBP chronicity shows that increased chronicity is associated with elevated MF FI and advanced disc degeneration at L4L5 and L5S1. (A) increased cLBP chronicity is associated with elevated mean deep15 MF FI%, higher mean disc degeneration at the lower lumbar levels, and with older age. (B) in the overall MF FI% model of chronicity, increased cLBP chronicity is associated with elevated mean overall MF FI% and with higher mean disc degeneration at the lower lumbar levels

cLBP Chronicity ~ mean deep15 MF FI% + mean DDD + Age + Sex + BMI			
	Estimate	SE	p-value
Intercept	−16.18	4.54	*p* <.001
mean deep15 MF FI%	0.17	0.05	0.002
mean DDD	2.04	0.72	0.005
AGE	0.14	0.04	0.002
SEX: F	−0.90	1.24	0.467
BMI	0.18	0.13	0.185
** cLBP Chronicity ~ mean overall MF FI% + mean DDD + Age + Sex + BMI**			
**Fixed Effect**	**Estimate**	**SE**	**p-value**
Intercept	−9.19	4.27	0.030
mean overall MF FI%	0.29	0.07	*p* <.001
mean DDD	2.18	0.70	0.002
AGE	0.07	0.05	0.180
SEX: F	−1.57	1.25	0.209
BMI	0.10	0.14	0.466

DDD: disc degeneration

### Modeling physical activity and its relationship to multifidus fat infiltration and cLBP chronicity

**Fig. 2:**
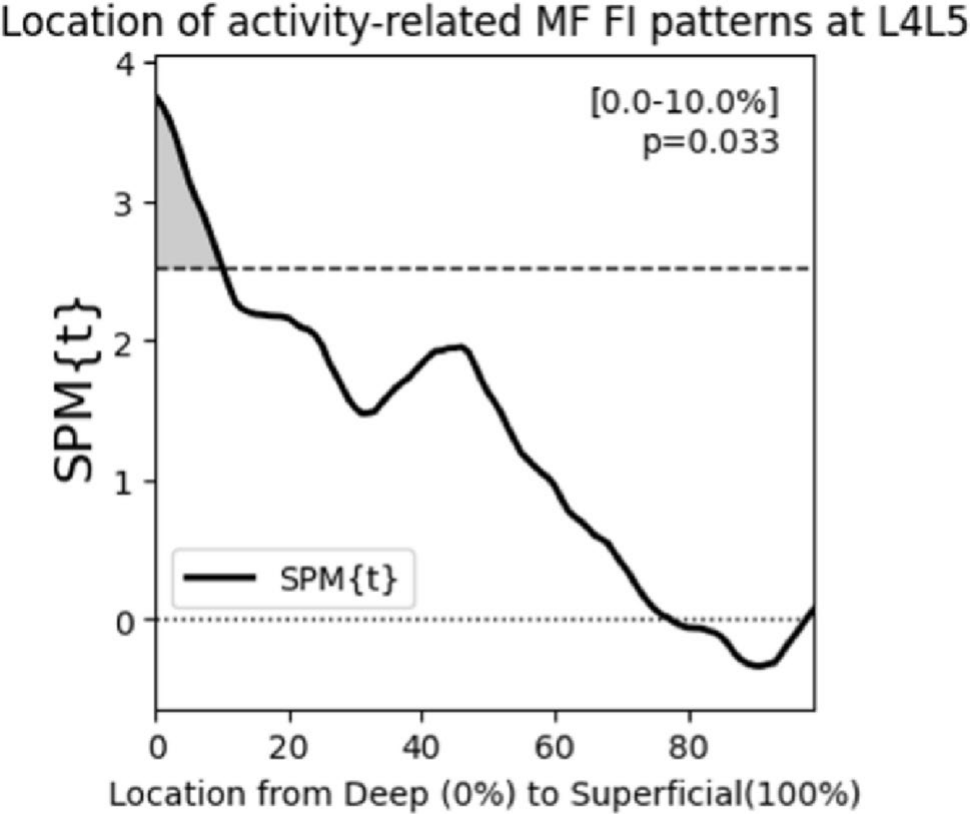
SPM general linear modeling shows that less physical activity associates with a pattern of elevated MF FI specifically in the deep MF at L4L5. Lower levels of physical activity are associated with elevated MF FI in the deepest 10% of the MF at L4L5. Physical activity levels did not associate with MF FI in any region of the MF at L5S1

Using a multivariable linear regression model of physical activity levels, we observed that physical activity levels did not associate with chronicity, pain intensity, age, sex, or BMI (Table 3). From SPM general linear modeling adjusting for age, sex, and BMI, we find that lower physical activity levels associated with higher MF FI in the deepest 10% of the MF at L4L5 (*p* <.05) (Fig. 2). At L5S1, there was no relationship between physical activity level and MF FI% in any region of the muscle.

**Table 3:** Linear regression of physical activity levels shows that physical activity levels in cLBP patients do not associate with pain severity, cLBP chronicity, age, sex, or BMI

Physical activity ~ cLBP Chronicity + Pain Severity + Age + Sex + BMI			
	Estimate	SE	p-value
Intercept	1846.66	1028.48	0.070
cLBP Chronicity	8.96	13.18	0.500
Pain Severity	−5.71	78.72	0.940
AGE	6.17	10.75	0.570
SEX: F	−518.07	303.17	0.090
BMI	45.20	34.75	0.190

Lastly, using a linear mixed-effects model of deep15 MF FI%, we find that longer duration of cLBP (*p* <.01) and lower physical activity levels (*p* <.05) associated with elevated deep15 MF FI%. Higher levels of disc degeneration (*p* <.001) and higher pain severity (*p* <.05) also associated with elevated deep15 MF FI%. In addition, deep15 MF FI% was higher in females (*p* <.001) and at L5S1 (*p* <.001) and increased with age (*p* <.001) but not with BMI. In the linear mixed-effects model of overall MF FI%, longer duration of cLBP associated with elevated overall MF FI% (*p* <.01). Physical activity level, pain intensity, and disc degeneration did not associate with overall MF FI%. However, overall MF FI% was higher in females (*p* <.001) and at L5S1 (*p* <.001) and increased with older age (*p* <.001) and higher BMI (*p* <.05) (Table 4).

**Table 4:** Linear mixed-effects modeling of multi-level MF degeneration shows elevated MF FI at L4L5 and L5S1 associates with increased cLBP chronicity, advanced disc degeneration, higher pain severity, and lower levels of physical activity. Elevated deep15 MF FI% was associated with increased cLBP chronicity, inactivity, increased pain intensity, and advanced disc degeneration. However, elevated overall MF FI% was associated with increased cLBP chronicity but not with physical activity levels, pain severity or disc degeneration. Further, higher BMI associated with higher overall MF FI% but not with deep15 MF FI%

Outcome ~ cLBP Chronicity + Disc Degeneration + Physical Activity + Pain Intensity + Age + Sex + BMI + Lumbar Level + (1|ID)						
Fixed Effect	Deep15 MF FI%			Overall MF FI%		
	Estimate	SE	p-value	Estimate	SE	p-value
Intercept	46.09	1.17	*p* <.001	22.12	0.84	*p* <.001
cLBP Chronicity	2.33	0.79	0.004	1.87	0.59	0.002
Physical Activity	−1.75	0.72	0.015	−0.81	0.54	0.130
Pain Intensity	1.61	0.72	0.027	−0.24	0.55	0.660
Disc Degeneration	2.12	0.64	*p* <.001	0.72	0.38	0.060
Age	3.13	0.78	*p* <.001	5.42	0.58	*p* <.001
SEX: F	6.62	1.45	*p* <.001	5.84	1.09	*p* <.001
BMI	0.55	0.73	0.46	1.3	0.55	0.02
Level: L5S1	11.45	0.98	*p* <.001	5.19	0.51	*p* <.001

## Discussion

Using a spatial approach to fat-map the MF adjusting for age, sex, and BMI, this study identified patterns of elevated FI in the deep and superficial MF associated with increased cLBP chronicity and reduced physical activity levels. Importantly, from linear mixed-effects modeling, we find that chronicity and physical activity levels are independently associated with MF FI. Further, these associations are independent of the adjacent disc degeneration and the patient’s reported pain severity. These findings are corroborated by linear regression modeling which shows that activity levels were not associated with cLBP chronicity or pain severity. This confirms that the association between lower physical activity levels and elevated deep15 MF FI% was not related to differences in duration or severity of cLBP symptoms.

Prior studies have shown that both older age and advanced disc degeneration are associated with elevated MF FI [9, 23, 24]. While we find that older age and advanced disc degeneration are also associated with increased cLBP chronicity, we show that cLBP patients with higher chronicity had higher MF FI even when accounting for age and disc degeneration, supporting the notion that MF FI is independently related to chronicity. In a prior study, the frequency of LBP episodes correlated with elevated PSM FI in recurrent LBP patients. Although we did not measure episode frequency, we find that cLBP patients with a longer history of cLBP have elevated MF FI at the lower lumbar levels independent of both age and disc degeneration. While some MF FI is attributable to aging and adjacent degenerative disc changes, cLBP persistence appears associated with MF degeneration through additional unknown factors. Limited to a cross-sectional analysis, it is unclear if the deep MF fatty infiltrates as cLBP persists or, inversely, if MF FI increases cLBP persistence.

Importantly, we do not observe a relationship between chronicity and physical activity levels, nor between physical activity levels and pain severity. Prior work has linked inactivity to increased skeletal muscle FI in healthy adults [25]. Further, physical activity has been shown to slow age-associated increase in skeletal muscle FI in older adults [17]. In the scope of cLBP, recent work has found an association between lower activity levels and higher PSM FI in both cLBP patients and controls [23]. Our results confirm this association in cLBP patients but only in the deep MF at L4L5. Similarly, a recent bed rest study found elevated levels of FI specifically in the deep regions of the MF at the lower lumbar levels [26]. Interestingly, physical activity levels did not associate with chronicity or pain severity. The elevated MF FI associated with decreased physical activity may be unrelated to lifestyle changes associated with duration or severity of pain.

This study’s strengths include the use of a spatial approach and advanced water-fat MRI sequences for accurate FI quantification and analysis. However, this study has notable limitations. First, although the IPAQ survey is a validated approach used in prior cLBP studies, questionnaires introduce recall bias and are more subjective than quantitative approaches like actigraphy. Similarly, we use a patient-reported measure for cLBP duration and only collect imaging cross-sectionally, limiting our ability to measure MF FI changes as cLBP persists. Lastly, we calculate FI% and create fat-maps using axial slices at the sagittal midpoint of the disc. Although we standardize this process across subjects, take the average of two slices, and distance normalize the radial fat-maps to standardize comparisons, we are unable to correct for differences in lumbar lordosis between subjects, which may alter the shape of the muscle presented in an axial slice. This limitation is not unique to fat-mapping, but a result of the axial slicing of the MRI.

Our findings have several important implications. First, a longer duration of cLBP is associated with MF degeneration beyond that associated with older age, and advanced disc degeneration, suggesting that other cLBP-related factors play a role in this association. It is unclear if cLBP persistence results in an accumulation of MF degeneration or conversely, poor MF quality may exacerbate cLBP persistence. Future work should explore the progression of MF degeneration in cLBP and the validity of MF FI measures as a marker of cLBP progression or a target for intervention. Second, while inactivity is associated with higher MF FI, differences in inactivity between patients appeared to be unrelated to pain severity or cLBP persistence. This suggests that the relationship between physical activity and deep MF quality is not the result of a lifestyle change driven by cLBP duration or severity. While this shows that physical activity levels independently associate with FI in the deep MF, it is unclear if physical activity may have an interventional role targeting deep MF FI in cLBP.

In summary, we report that increased cLBP chronicity associates with elevated MF FI independent of age, disc degeneration and physical activity level. Importantly, differences in activity between patients were not related to chronicity or pain severity. Lower physical activity levels independently associated with elevated MF FI specifically in the deep MF, where fat infiltration has been linked to cLBP symptoms and adjacent disc degeneration. Future work should examine whether PSM health is a mechanism by which physical activity serves as an intervention for cLBP.

## Data Availability

The data that support the findings of this study are not openly available to protect study participant privacy.
